# Effect of highly concentrated bleaching gels on enamel microhardness and superficial morphology, and the recovery action of four remineralizing agents

**DOI:** 10.1186/s12903-022-02693-2

**Published:** 2022-12-27

**Authors:** María Melo, Rossella Fioresta, José Luís Sanz, María Pilar Pecci-Lloret, Carmen Llena

**Affiliations:** 1 Departament of Stomatology, Universitat de València, València, Spain; 2 Special Care and Gerodontology Unit, Department of Dermatology, Stomatology, Radiology and Physical Medicine, School of Dentistry, University of Murcia, 30008 Murcia, Spain

**Keywords:** Enamel microhardness, Enamel morphology, Remineralization, Hydrogen peroxide, Dental bleaching

## Abstract

**Background:**

Dental bleaching is a common clinical practice. The aim of this study is to investigate the effect of 35% hydrogen peroxide (HP) bleaching gel on the morphology and microhardness of enamel, and to analyze the effect of four remineralizing agents.

**Methods:**

One hundred blocks were prepared. The enamel surfaces were bleached with 35% HP in one session. The specimens were divided into four remineralization treatment groups (n = 25). G1: Tooth Mousse, G2: Remin-Pro, G3: Colgate Pro-Relif, G4: Mirafluor. The remineralizing protocol was applied 3 min per day for one week. Vickers microhardness (HV) measurements and SEM observations were performed at baseline, after bleaching, and after remineralizing treatment in all groups. Statistical analyses were performed using the paired t-test and ANOVA.

**Results:**

After bleaching, SEM showed an increase of irregularities on the surface of the samples. Enamel microhardness decreased a mean of 47.7 HV, equivalent to a mean decrease of 18.3% (*p* < 0.05). After remineralization, the HV increased in all groups between 16 and 33% (*p* < 0.01), recovering the initial microhardness of enamel samples. SEM images revealed a higher quantity of superficial mineral deposits in groups 1 and 2 compared to the rest of the groups.

**Conclusions:**

The application of remineralizing products generates a significant increase in enamel microhardness. Tooth Mousse-treated samples showed a greater microhardness recovery, followed by Remin Pro. The superficial morphology of the samples reflects the results obtained in the HV tests.

## Background

The interest in dental bleaching has grown in recent years, both for patients, clinicians, and researchers. The current aesthetic reference is represented by light-toned, well-shaped, and correctly aligned teeth. With an adequate diagnosis and treatment planning, bleaching can represent a safe and conservative technique to manage dental discolorations [1–3].

Contemporary bleaching systems are mainly based on the use of hydrogen peroxide (HP) directly or indirectly released from other products, such as carbamide peroxide (PC) [4].

The permeability of enamel and dentin and the low molecular weight of bleaching agents aid their penetration [5]. As previously reported in the literature, bleaching leads to changes in the enamel structure regarding three aspects: mineral loss, changes in surface morphology, and alteration of surface microhardness [6–8]. These changes are related to the concentration of the product and the exposure time [9–11].

Saliva and other active agents play an essential role in creating an environment that reduces demineralization and promotes remineralization in enamel exposed to bleaching agents [12]. Several studies have described that the use of remineralizing products after whitening procedures could repair the morphological defects and mineral loss induced by the whitening agent, due to their chemical similarity with the mineral component from the teeth [13–17].

The superficial application of fluoride results in the formation fluorapatite on the enamel surface, recovering its hardness and promoting remineralization [18]. However, some authors have reported a lack of improvement after the incorporation of fluoride on bleached enamel [19], or even a lack of significant morphological changes after the application of acidified fluoride gel after a bleaching treatment with 35% hydrogen peroxide [20]. It should be noted that topical fluoride varnishes promote several biological responses on human gingival cells, so the application of these varnishes must be done carefully.

Casein phosphopeptide-amorphous calcium phosphate (CPP-ACP) and casein phosphopeptide-amorphous calcium fluoride phosphate (CPP-ACFP) are two nanocomplexes that act as reservoirs of calcium and phosphate (and fluoride in the case of the latter). Prior to an acid exposure they release these ions, resulting in the generation of an oversaturated environment which favors remineralization [21]. When applied after dental bleaching, the remineralization and microhardness of enamel is increased [15, 22, 23].

Another agent with remineralizing properties is arginine, an amino acid that is positively charged at physiological pH which acts as a pH buffer and as a source of calcium. It is currently commercially-available for the treatment of dentinal hypersensitivity and has shown to improve surface morphology and increase enamel microhardness in teeth undergoing whitening [12, 24, 25]. However, there are few studies in the literature that discuss the effects of arginine-containing toothpastes to prevent or reverse the effects that may occur before, during, or after bleaching [12, 24, 25].

Remin Pro is another remineralizing agent that contains calcium and phosphate in the form of hydroxyapatite, fluoride, and xylitol. Hydroxyapatite fills the spaces of damaged enamel, fluoride seals the dentin tubules, and xylitol acts as an antibacterial agent. Some authors argue that this product is suitable for treating dentin hypersensitivity, preventing enamel demineralization and promoting remineralization of enamel lesions, thus improving its properties after the application of bleaching substances [26].

The availability of a wide variety of remineralizing products in the market and the increasing popularity of bleaching as a treatment of dental discolorations results in the need to investigate their potential combined use. Accordingly, the objective of the present in vitro study is to evaluate the effect of commercially-available remineralizing products on the superficial morphology and the microhardness of enamel, after being treated with a high-concentration hydrogen peroxide. Two null hypotheses were established: a) The application of a high-concentration bleaching agent does not affect the properties of enamel in terms of micro-hardness and surface morphology; and (b) The superficial application of remineralizing products on bleached enamel produce no variation in its microhardness or superficial morphology.

## Methods

### Experimental design

The following experimental protocol was approved by the ethics committee of University of Valencia (procedure number 1208761).

50 teeth (premolars and molars) extracted for periodontal reasons, free of pigmentation, cracks, structural defects, or caries lesions were used. Visual inspection of the potentially eligible teeth was performed under an optical microscope (40× magnification). The selected teeth were stored in distilled water at a constant temperature of 25 °C until the beginning of the study.

The buccal and palatal/lingual surfaces of the samples were cleaned using a pumice stone under low-speed rotation and water cooling. The teeth were then horizontally sectioned at the level of the cementoenamel junction (CEJ) using a laboratory trimmer (Struers, Copenhagen, Denmark) and a diamond disc (Struers, Copenhagen, Denmark) at 400 rpm. Subsequently, a second cut was made in the crown in a mesiodistal direction, separating the mesial and distal surfaces. Lastly in a third cut in a buccolingual direction, the buccal surface was separated from the palatal/lingual. A total of 100 samples with 3 × 3 × 3mm (± 0.25 mm) dimensions were obtained.

**Fig. 1:**
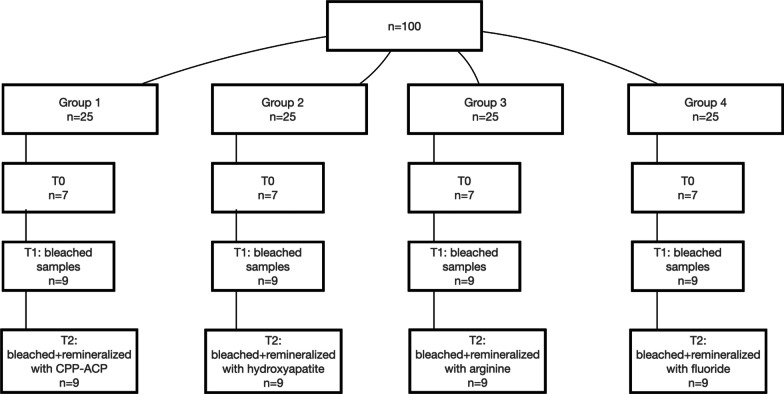
Sample distribution. Groups and subgroups

After the cuts were performed, samples were washed with distilled water for 30 s to remove debris, dried with a sterile gauze, and nail varnish was applied to the dentin margins to prevent leakage of the bleaching agent. The 100 samples were randomly divided into 4 groups (n = 25) using simple random sampling. Each group was further subdivided into three test groups, G0, samples that did not receive any type of treatment (n = 7); G1, samples submitted to the bleaching protocol (n = 9); and G2, samples submitted to the bleaching protocol and subsequent remineralization (n = 9). The distribution of the samples is shown in Fig. 1

The samples were stored in artificial saliva (Table 1), at a constant temperature of 25°, which was changed daily until the beginning of the bleaching protocol.

**Table 1:** Chemical composition of saliva

Components	Concentration g/l
KCl	0.963 g
NaCl	0.125 g
Urea	0.2 g
CaCl_2_–2h_2_O	0.227 g
KH_2_PO_4_	0.654 g
NaHCO_3_	0.63 g
Agua destilada	1000 ml

### Bleaching protocol

Seventy-two samples (18 samples per group, belonging respectively to subgroups G1 and G2) were subjected to the following bleaching protocol:

All samples were dried with a sterile gauze and the bleaching agent was applied, which was 37.5% HP in a gel with a neutral pH (PolaOffice; SDI, Victoria, Australia; lot number: 1137287). Bleaching was performed in single session, with 4 8-min applications. Between applications, the bleaching agent was removed with sterile gauze. Once the established bleaching protocol had been carried out, the samples were rinsed with distilled water for 30 s and stored in artificial saliva for 24 h.

### Remineralization protocol

A total of 36 samples were subjected to the post-whitening remineralization protocol, representing 9 samples per group (G2).

A different remineralizing product was applied to each group; group 1: CPP-ACP (Tooth Mousse, Gc, Tokyo, Japan); group 2: Remin-Pro (Voco, Cuxhaven, Germany); group 3: 8% Arginine (Colgate sensitive Pro-Relief); group 4: fluoride (Mirafluor C, Miradent, Germany,). All products were applied 3 min per day using a microbrush, for 7 days. After each application, the samples were dried with a sterile gauze and kept in artificial saliva that was renewed daily. The chemical composition of the products used in this study is reflected in Table 2.

**Table 2:** Chemical composition of remineralizing agents used

Materials	Chemical composition	Manufacturer
CPP-ACP Tooth mousse	Pure water, glycerol, CPP-ACP, D-sorbitol, CMC-Na, propyleneglycol,silicondioxide, titanium dioxide, xylitol, phosphoric acid, flavoring, zincoxide,sodiumsaccharin, ethyl. p-hydroxybenzoate, magnesiumoxide, guargum, propylp-hydroxybenzoate, butyl p-hydroxybenzoate	GC Corporation, Tokyo, Japan
Hidroxiapatite Remin-Pro	Sodiumfluoride, ethanoliccolophony, Hydroxyl apatite Xylitol	VOCO GmbH,Cuxhaven, Germany
Arginine 8% Colgate sensitive Pro-Relife	Arginine 8%, calcium carbonate, glycerin, water, bicarbonate, cellulose gum, Hydrated silica, sodium saccharin, Sorbitol, Sodium Lauryl Sulfate, Sodium Monofluorophosphate (1450 ppmF), Aroma,Sodium Silicate, Sodium Bicarbonate, Titanium Dioxide, Potassium Acesulfame, Xanthan Gum,Sucralose	Colgate-Palmolive, Poland
Fluoride 1,23% Mirafluor C	Aminofluoryd 1,25%,Water,Sorbitol,Hydrated Silica,Propylene Glycol,Olaflur,Cocamidopropyl,Betaine,Aroma,Hydroxyethylvellulose,Sodium Saccharin,Sodium Chloride,Limonene,CI77891	Miradent, Germany

### Vickers microhardness (VHN) measurement

Three microhardness measurements were performed: baseline, before starting the experimental protocol (T0); 24 h after applying the bleaching protocol (T1); and 24 h after completing the remineralization protocol (T2). The VHN values were determined using a Duramin microdurometer (Struers, Denmark) with a load of 100 g for 15 s. Three measurements were made for each sample in randomly selected areas on the enamel surface and the mean values were subsequently determined.

### Morphological visualization under scanning electron microscopy (SEM)

**Fig. 2:**
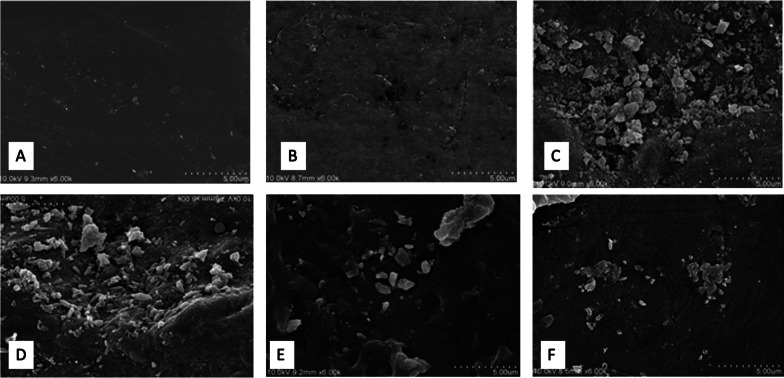
**A**: untreated enamel surface observed under 6000× magnification. **B**: Enamel surface subjected to bleaching (6000× magnification). **C**: Distribution of minerals on the enamel surface bleached and remineralized with Tooth Mouse (6000× magnification). **D**: Distribution of minerals on the enamel surface bleached and remineralized with Remin-Pro (6000× magnification). **E**: Distribution of minerals on the enamel surface bleached and remineralized with Remin-Pro (6000× magnification). **F**: Distribution of minerals on the enamel surface bleached and remineralized with Mirafluor C (6000× magnification)

All samples were fixed on an aluminum platform, processed with a palladium gold bath for 2 min and visualized under SEM (S-4100 scanning electron microscope; Hitachi Tokyo, Japan) using a power of 10 kV, at 6000×, 50000×, and 100,000× magnifications. Relevant areas were selected and their surface morphology was examined, identifying different areas according to the following classification: 0: enamel with smooth surface morphology, 1: enamel with slight irregularities, 2: enamel with moderate irregularities, 3: enamel with accentuated irregularities; in the case of untreated (G0) and bleached samples (G1) (Fig. 2).

In the case of samples treated with remineralizing agents (G2), the presence of mineral deposits was recorded as follows: 1: presence of slight mineral deposits, 2: presence of moderate mineral deposits, 3: presence of accentuated mineral deposits [20].

### Statistical analysis

The normal distribution of the hardness measurements for the different test groups at T0, T1, and T2 in the groups was evaluated using the Kolmogorov–Smirnov test, with confirmatory results (*p* > 0.05). Accordingly, the statistical approach was parametric. However, given the sample size of the subgroups, alternative non-parametric models were performed to ensure the robustness of the results. An inferential analysis was also performed, consisting of the estimation of a repeated measures t-test to evaluate the changes in mean microhardness in the complete sample from T0 to T1 (bleaching effect). To check the homogeneity of the previous effect in the defined groups, the previous t-test was generalized into a General Linear Mixed Repeated Measures Model for the Vickers microhardness response variable, whose design includes an intra-subject factor or repeated measures: the time (T0, T1), and the group (1/2/3/4). Bonferroni multiple comparison tests were applied to assess differences between groups within a time or between times within a group. The same was performed for the analysis of the remineralizing effect in the T1–T2 phase. The model was also replicated to evaluate the changes from T0 to T2, evaluating whether the complete protocol (bleaching + remineralization) induced any difference in the level of hardness. As an alternative non-parametric method, a Brunner-Langer model was estimated for longitudinal data, estimating the same effects by means of an ANOVA-type ATS test. The level of significance used in the analyzes was 5% (α = 0.05). For an ANOVA model such as the one described, with a significance level of 5% and considering an effect size of f = 0.25 (mean) to be detected, the power reached is 0.99 for the contrast of the intra-subject effects and 0.65 for inter-subject effects.

After the morphological analysis of the samples via SEM, the percentage of samples for each degree of affectation was calculated, and the presence of mineral deposits after the application of the different remineralizing agents was compared by the chi-squared test. Statistical significance was considered as *p* < 0.05.

## Results

### VHN measurement

At T0, the mean microhardness was 260.2 ± 85.1 HV, with 95% CI (243.3–277.1). At T1, the mean value decreased to 212.5 ± 67.5 HV, with 95% CI (199.1–225.9). Therefore, the loss of hardness due to the bleaching action was 47.7 ± 60.1 or, in relative terms, − 18.3%. The result of the t-test concluded that the bleaching procedure resulted in a significant loss of microhardness (*p* < 0.001).

The ANOVA model concluded that the bleaching treatment generated a reduction in hardness in the sample as a whole. The most relevant aspect of the model is that the loss of hardness is similar between the groups (*p* = 0.941).

Analyzing the effects of the remineralization treatment, the recovery of hardness took place in the 4 groups and in an approximately similar magnitude. The highest recovery was observed in group 1 (Tooth Mousse), and the least was exhibited by group 4 (Mirafluor C).

As a percentage, the average gain of the 4 groups was 32.7%, 26.1%, 23.0% and 15.9%, respectively. The ANOVA model concluded a general recovery of hardness from T1 to T2 (*p* < 0.001), which was similar in the 4 groups (*p* = 0.186). No differences were observed between T0 (pre-whitening) and T2 (post-remineralization) (*p* = 0.476), meaning that the entire process returned the hardness to basal levels. This result can be extrapolated to the 4 groups (*p* = 0.330). However, in group 1 (Tooth Mousse), the differences are close to statistical significance (*p* = 0.081), exhibiting final hardness value which were markedly higher than the initial ones.

### SEM visualization

The adamantine surfaces of the teeth, observed before carrying out any treatment (T0), were seen to be uniform or with small irregularities. Accordingly, samples were classified as grade 0: enamel with smooth surface morphology (Fig. 2A), and grade 1: enamel with slight irregularities. In the samples subjected to the bleaching protocol, significant changes were observed in the adamantine layer of the samples, exhibiting depressions and irregularities. Consequently, they were classified as grade 2: enamel with moderate irregularities, and grade 3: enamel with marked irregularities (Fig. 2B).

After the remineralizing treatment, amorphous mineral deposits were observed on the enamel surface in all groups to a greater or lesser extent. All samples from the group treated with GC Tooth Mousse showed clear signs of remineralization. These samples were classified as grade 3 (Fig. 2C). Clear signs of remineralization could also be observed in the samples treated with Remin-Pro. These samples were also classified as grade 3 (Fig. 2D). In samples treated with Sensitive Pro-Relief and Mirafluor C, enamel defects caused by bleaching remained noticeable and few mineral deposits were visible. The enamel surfaces of these groups were categorized as grade 1 (Fig. 2E, F).

The presence of deposits after the application of remineralizing agents was significantly higher in the groups treated with GC Tooth Mousse, Reminpro, and Mirafluor (100% of the samples showed accentuated mineral deposits); while in the arginine group, only slight mineral deposits were found among the samples (*p* < 0.05).

## Discussion

Similar studies among the literature are not homogeneous in terms of the origin of the substrate (i.e. human or bovine,) sample preparation, sample size, storage conditions between treatment intervals (without remineralizing solution, with remineralizing solution, stored in artificial saliva or stored in distilled water), and type of tests to evaluate microhardness (Knoop vs. Vickers hardness) [13–16].

In this study, 50 human teeth extracted for periodontal reasons were used, as performed by similar studies in the field [5, 8, 15, 20, 27, 28]. The buccal and palatal/lingual surfaces of the teeth were used for the study, obtaining a total 100 standardized samples [29–32]. The surfaces of the samples were cleaned with a pumice stone under low-speed rotation and refrigeration, aiming to reproduce the clinical conditions as closely as possible without altering the surface of the samples [5, 16]. Some authors, however, treat sample surfaces by polishing in order to obtain samples with smooth surfaces [15, 27, 29, 31, 33–36]. In the present study, all samples were stored in artificial saliva, changed daily, as proposed by other authors [16, 27, 28, 30, 31].

Bleaching was carried out in a single session with 4 8-min applications of HP at 35%, as proposed by the manufacturer. Although some protocols propose repeating the treatment session in successive weeks [25, 33, 35], a single session was chosen for the present study, since in clinical practice it is relatively common for several applications to be made in a single session.

Once the bleaching was performed, the remineralization protocol was applied following the manufacturers' instructions, contrary to other authors, who establish a single application after bleaching [5, 27].

Vickers microhardness tests were carried out by applying a force of 100 g for 15 s, as performed previously in other studies [14, 30, 33]. Three measurements were made and the mean value of each measurement was considered, at baseline (T0), after bleaching (T1), and after applying the remineralization protocol (T2) [14, 27, 32, 36, 37]. This was performed with the objective of determining the effects of the bleaching agent on the microhardness of the enamel and the ability of the remineralizing products to restore the initial conditions of the enamel. These conditions can be different according to the patients’ diet.

The first null hypothesis was rejected, since despite the fact that tooth bleaching is considered one of the most conservative treatments, we must not ignore the changes that occur on the enamel surface that result in a reduction in its microhardness caused by mineral loss [8, 38]. This was confirmed by the results obtained in the present study, in which a significant reduction in the microhardness in all specimens exposed to 35% HP bleaching treatment was observed. This result is similar to that reported by other authors, who also observed a significant reduction in enamel microhardness after bleaching with high-concentration HP, regardless of its pH. In the present study, PolaOffice, a neutral pH gel [10], was used.

It has been previously described that the effect of these bleaching gels can be reversed with the application of different remineralizing agents [25, 27, 29, 30, 39]. This was confirmed in the present study, in which all samples restored their microhardness to a level similar to that found before bleaching. Therefore, the second null hypothesis was also rejected. The recovery of hardness took place in all the groups and in an approximately similar magnitude. This recovery was found to be highest in the group to which CCP-ACP was applied, followed by the group exposed to hydroxyapatite and Arginine 8%, and to a lesser extent in the group exposed to 1.23% fluoride. These results coincide with those obtained by other authors [22, 29, 30], who concluded that the application of CCP-ACP contributes to recovering the mechanical properties of enamel. On the other hand, Soares et al. [14], in their study based on the microhardness analysis of four groups subjected to a demineralization protocol and successively exposed to the action of four remineralizing products, observed that the highest microhardness values post-remineralization were exhibited by the p11 peptide-exposed group, followed by the CPP-ACP-exposed group and by bioactive glass, while the lowest values were observed in the hydroxyapatite-exposed group. Bayrak et al. [22] concluded that the groups exposed to bleaching and subsequently to the application of CCP-ACP or CCP-ACPF showed a post-treatment microhardness which was significantly higher than the baseline microhardness (*p* < 0.001). These authors also concluded that the group subjected to remineralization with 1.23% fluoride obtained microhardness values lower than those found with casein phosphopeptides with calcium and amorphous phosphate.

The remineralizing effect of CPP-ACP is due to the fact that casein phosphopeptide has a remarkable ability to stabilize calcium phosphate in the form of ACP nanocomplexes, thus allowing the formation of small CPP-ACP clusters. These CPP-ACP groups act as a deposit of calcium and phosphate that adheres to tooth surfaces and dental plaque. Upon exposure to an acidic environment, CPP-ACP releases calcium and phosphate ions, thus maintaining a supersaturated environment, which reduces demineralization and facilitates remineralization [40].

Regarding the surface morphology of the samples from the studies conducted by Soares et al. [14] and Coceska et al. [5], in the group that only underwent bleaching, areas of damaged enamel and other areas of intact enamel were observed, a result that coincides with what was obtained in our study. In the case of the study carried out by Soares et al. after the application of the remineralizing products, especially in the CPP-ACP group, remineralization of the surface was observed, which was manifested by the deposit of crystals around the enamel prisms. Coceska et al. [5] concluded that all samples treated with CCP-ACP showed signs of remineralization through the presence of amorphous deposits, although areas of damaged enamel and loss of integrity were still visible. The groups treated with 1.23% fluoride also presented enamel areas with depressions and erosion caused by the bleaching agent, with few signs of remineralization. Poggio et al. [28] observed that the group that had only undergone bleaching presented an irregular and porous surface, whereas the groups exposed to remineralizing products showed an improvement in surface morphology, with the CPP-ACP and hydroxyapatite group standing. The images of the enamel surface of the samples treated by CPP-ACP were more regular compared to those treated with fluoride-based pastes. Vieira-junior et al. [12] in their study concluded that the enamel surface treated with HP showed irregularities, and that the groups treated with 8% Arginine or Novamin presented mineral precipitates deposited in the areas demineralized by the bleaching agent. Altogether, these results are consistent with those of our study.

## Conclusions

The application of hydrogen peroxide resulted in a significant decrease in enamel microhardness. The application of remineralizing agents generated a significant increase in microhardness, recovering the initial microhardness of the enamel before bleaching treatment. From a morphological point of view, the products based on CPP-ACP and hydroxyapatite showed a greater amount of surface deposits than those based on 8% arginine or fluoride.

## Data Availability

The datasets used in this study are available from the corresponding author on reasonable request.

## Abbreviations

HP: Hydrogen peroxide
PC: Carbamide peroxide
SEM: Scanning electron microscopy
VHN: Vickers microhardness measurement
